# Molecular Aspects of Varicella-Zoster Virus Latency

**DOI:** 10.3390/v10070349

**Published:** 2018-06-28

**Authors:** Daniel P. Depledge, Tomohiko Sadaoka, Werner J. D. Ouwendijk

**Affiliations:** 1 Department of Microbiology, New York University School of Medicine, New York, NY 10016, USA; 2 Division of Clinical Virology, Center for Infectious Diseases, Kobe University Graduate School of Medicine, 7-5-1 Kusunoki-cho, Chuo-ku, Kobe 650-0017, Japan; tomsada@crystal.kobe-u.ac.jp; 3 Department of Viroscience, Erasmus Medical Centre, 3015 CN Rotterdam, The Netherlands; w.ouwendijk@erasmusmc.nl

**Keywords:** varicella-zoster virus, latency, reactivation, sensory ganglia, VZV latency-associated transcript, open reading frame 63, RNA-sequencing, epigenetics, immunity

## Abstract

Primary varicella-zoster virus (VZV) infection causes varicella (chickenpox) and the establishment of a lifelong latent infection in ganglionic neurons. VZV reactivates in about one-third of infected individuals to cause herpes zoster, often accompanied by neurological complications. The restricted host range of VZV and, until recently, a lack of suitable in vitro models have seriously hampered molecular studies of VZV latency. Nevertheless, recent technological advances facilitated a series of exciting studies that resulted in the discovery of a VZV latency-associated transcript (VLT) and provide novel insights into our understanding of VZV latency and factors that may initiate reactivation. Deducing the function(s) of VLT and the molecular mechanisms involved should now be considered a priority to improve our understanding of factors that govern VZV latency and reactivation. In this review, we summarize the implications of recent discoveries in the VZV latency field from both a virus and host perspective and provide a roadmap for future studies.

## 1. Introduction

Most adults worldwide are infected with the neurotropic human alphaherpesvirus, varicella-zoster virus (VZV) [1]. VZV is the causative agent of two distinct diseases: a generalized vesicular skin rash referred to as varicella (chickenpox), and a localized dermatomal skin rash referred to as herpes zoster (HZ; shingles) [2]. Although varicella and HZ were known to be related [3], it was not until 1965 that the British general practitioner, Dr Robert E. Hope-Simpson, suggested that “*herpes zoster is a spontaneous manifestation of varicella infection*”. This observation was based on a careful examination of ~3500 patients, including 192 HZ cases, who visited his practice over a 16-year period, combined with cautious reading of the available anatomical and epidemiological literature regarding HZ. This led to his famous hypothesis that: “*Following the primary infection (chickenpox), virus becomes latent in the sensory ganglia, where it can be reactivated from time to time (herpes zoster)*” [4]. Eighteen years later, this hypothesis was proven by Dr Donald Gilden’s crucial discovery of VZV DNA in latently infected human ganglia [5]. Although we have learned much about VZV biology in general in previous decades, the mechanisms underlying VZV latency and reactivation have remained enigmatic, at least in part due to VZV being a human tropic pathogen that does not cause disease in experimental animal models. However, recent insights into the VZV latency program and newly developed in vitro models using human embryonic stem cell (hESC)-derived neurons for studying viral latency and reactivation have now set the stage to unravel the molecular mechanisms that regulate VZV latency. In preparing this review, we sought to be inclusive of as many view points as possible but to consider only human-derived cell- or tissue-based systems (animal models utilised for VZV studies are the subject of another review in this issue). At the outset, we therefore limit this review to only include studies of VZV latency in which there is a demonstrable lack of infectious virus production and/or any observable pathology.

## 2. Molecular Biology of VZV

### 2.1. Structure and Genomic Organization of VZV

**Figure 1:**
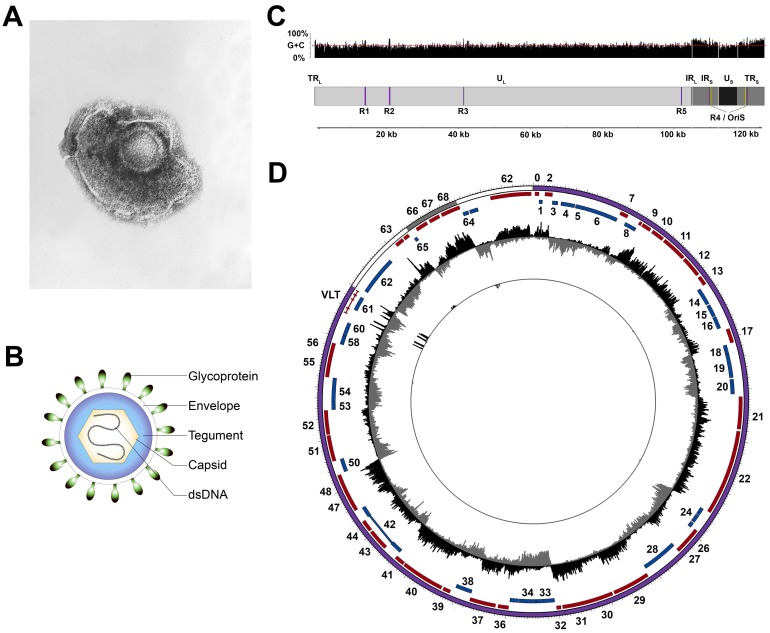
Structure of varicella-zoster virus (VZV) particles and genome. (**A**) Electron microscopy image of VZV (obtained from Centers for Disease Control and Prevention (CDC)/Dr Erskine Palmer; B.G. Partin. CDC Public Health Image Library). (**B**) Schematic representation of the VZV virion. (**C**) Schematic representation of the VZV genome structure and G + C content. (**D**) The VZV transcriptome profile during lytic infection of ARPE-19 cells (outer track) and latent infection of human TG (trigeminal ganglia; inner track). Note that the low frequency of VZV-infected neurons in human TG necessitates the use of targeted enrichment of VZV transcripts to detect VZV latency-associated transcript (VLT) expression [27]. Circos plot of the VZV genome (U_L_, TR/IR, and U_S_ are shown as purple, white, and grey bands, respectively; sense and antisense open reading frames (ORFs) are indicated as red and blue blocks, respectively, with ORF numbers indicated where possible). Data represent strand-specific VZV-enriched mRNA-sequencing with peaks facing outwards from the center (black) indicating reads mapping to the sense strand, while peaks facing inward (grey) originate from the antisense strand. The *y*-axis is scaled to the maximum read depth per library in all cases. dsDNA, double-stranded DNA; U_L_, unique long; U_S_, unique short; TR, terminal repeat; IR, internal repeat; R, reiterative region; Ori, origin of replication.

VZV particles are pleomorphic to spherical in shape, ~150–200 nm in diameter and are composed of three proteinous layers: a nucleocapsid containing the viral double stranded DNA (dsDNA) genome, a tegument layer, consisting of numerous proteins of both viral and host origin surrounding the nucleocapsid, and an envelope comprising a host-derived lipid bilayer inserted with viral glycoproteins facing outwards (Figure 1A,B) [6,7,8]. Upon entry of a VZV virion into the host cell, tegument proteins are released into the newly infected cell, altering the host environment, thereby inhibiting antiviral responses and influencing the fate of the virus program, i.e., a lytic or latent infection (reviewed in ref. [9]). The VZV dsDNA genome is about 125 kilo base pairs (kbp) in size and has a G + C content of 46%. It is composed of two unique segments, termed unique long (U_L_) and unique short (U_S_), that are flanked by inverted terminal repeat (TR) and internal repeat (IR) structures with high G + C contents (68% for the TR_L_/IR_L_ and 59% for the IR_S_/TR_S_) (Figure 1C) [10,11,12,13,14,15,16,17,18,19]. The very short (~88 bp) TR_L_ and IR_L_ sequences flank the U_L_ region, while the long (7319 bp) IR_S_ and TR_S_ sequences flank the U_S_ region. The composite structure generally allows for two isomeric configurations [17] that differ only in terms of whether the U_S_ region is inverted. While up to 5% of virions are reported to contain structural isoforms with an inverted U_L_ region [20], their relative scarcity is attributed to a unique DNA sequence at the extreme 5′ end of the U_L_ region that is required for viral DNA cleavage during packaging [21] i.e., these virions may not contain functional genomes. Five regions of the genome contain tandem direct reiterations (R1, R2, R3, R4, and R5) of short repeat sequences, one of which (R4) is located in the IR_S_/TR_S_. All except R5 are G + C rich, and all are subject to length and structural polymorphisms that vary both within and between strains [22,23,24,25,26]. Three of these reiterative regions (R1, R2, and R3) are located within the coding portion of VZV genes (*open reading frame* (*ORF*) *11*, *ORF14*, and *ORF20*, respectively) and may therefore exert an effect on protein function. Two copies of R4 are present in the IR and TR, neighboring the origin of replication (OriS), while R5 is located between *ORF60* and *ORF61*.

### 2.2. Coding Potential of the VZV Genome

The VZV genome was originally reported to encode 65 unique viral genes, three of which are located in the duplicated IR_S_/TR_S_ region [19]. Four additional VZV genes have since been identified including *ORF0* [28], *ORF9A* [29], *ORF33.5* [30], and the newly discovered VZV latency-associated transcript (*VLT*) [27] (Figure 1D). An underappreciated feature of VZV is that transcription of several genes, including *ORF0*, *ORF42/45*, *ORF50*, and *VLT*, require the host-splicing machinery to remove introns from pre-mRNA and have also shown evidence of alternative splicing, resulting in the synthesis of alternative proteins [19,27,31,32,33]. It thus seems likely that the full transcriptional potential of VZV has yet to be revealed, and we predict that the latest technological advances (e.g., full length sequencing of native RNA [34]) will yield further novel discoveries. It is also worth noting that the encoding of additional RNA types, including microRNAs and small non-coding RNAs, is still an area of active study with contrasting results [27,35,36]. How these studies might have an impact on our understanding of viral latency remains to be seen.

### 2.3. VZV Gene Expression during Productive Infection

Based on limited experimental data [37,38], and mostly by analogy to other *alphaherpesviruses*, VZV genes have been loosely designated as immediate-early (IE) genes, early (E) genes, and late (L) genes, with each ordered wave of expression thought to be dependent on the protein products of previous classes. Proteins encoded by IE genes act as transcriptional regulators, while those produced by E genes are mainly involved in DNA replication, and L genes encode structural proteins that are crucial for virion formation and egress. VZV encodes at least five transcriptional regulatory proteins specified by four putative IE genes, *ORF4*, *ORF61*, *ORF62* and *ORF63*, and one L gene, *ORF10*. All except the ORF61 protein, IE61, are part of the VZV virion [39,40]. Our understanding of the transcriptional regulation of VZV genes remains incomplete, in part due to the high cell-associated nature of VZV that precludes synchronized infections using cell-free viruses. The dominant transcriptional regulator and possibly only true immediate-early protein encoded by *Varicellovirus* is homologous to VZV IE62 [39,41,42,43,44,45,46,47,48]. Consistent with this idea, the VZV IE62 major viral transactivator protein can activate all three kinetic classes of VZV genes in the absence of other viral proteins, including all IE genes, *ORF4*, *ORF61*, *ORF62*, and *ORF63*, while IE4, IE61 and IE63 either do not or minimally stimulate the *ORF61* promoter ([49,50,51], reviewed in ref. [52]). Host transcription factors, either by themselves or through interactions with viral transcriptional regulatory proteins, also contribute to viral gene expression (reviewed in ref. [52]). VZV virion proteins delivered into newly infected cells upon entry are not absolutely required to initiate VZV gene expression, as evidenced by the resulting VZV replication upon transfection of cells with viral DNA (reviewed in ref. [53]). Notably, near identical VZV transcriptomes are detected during productive infection of diverse cell types, including neurons [27,54,55,56,57], suggesting a prominent role for either commonly expressed cellular transcription factors or viral proteins in coordinating VZV gene expression.

### 2.4. Stability of the VZV Genome

Recent advances in the fields of genomics and computational biology have led to an explosion in high-throughput VZV genome sequencing studies [25,58,59,60,61,62,63], with multiple studies focused on exploring the evolution of VZV [60,64,65]. Perhaps the most pertinent observations made by these studies are that (i) the VZV genome is very stable with over 98% sequence conservation between the most distant strain sequences to date, and (ii) the evolutionary history of VZV, like other herpesviruses, is shaped by extensive recombination [60], the latter requiring that two or more different viral genomes occupy the same cell nucleus during viral DNA replication. The live-attenuated nature of the VZV vaccine has enabled comparative analyses of vaccine-induced varicella and HZ isolates, showing that the VZV genome remains highly stable during latency [59,62].

## 3. Location of Latent VZV

### 3.1. Sites of VZV Latency

During primary infection, VZV infects and establishes lifelong latency in sensory neurons located in the dorsal root ganglia (DRG) and trigeminal ganglia (TG). While VZV DNA has been detected in various other sensory (geniculate, vestibular, and spiral ganglia) [5,66,67,68] and autonomic ganglia (nodose, enteric, and thoracic sympathetic ganglia) [69,70,71], it remains unclear as to whether the virus can establish latency and reactivate from all of these sites, i.e., infection of these sites may lead to the persistence of only VZV genomes that are not capable of reactivating. The only confirmed sites at which VZV can establish latency and reactivate are the DRG and TG, where the latent viral DNA is maintained as a circular episome in ~2–5% of sensory neurons at a median of 5–7 genome copies per neuron [72,73,74,75]. Additionally, VZV DNA and a restricted number of viral transcripts have been detected in intestinal biopsies of naturally infected and vaccinated individuals [69,76,77,78]. Although in situ detection of VZV DNA or RNA in human enteric neurons is lacking, clinical evidence suggests that VZV reactivation from the enteric nervous system (ENS) could be associated with gastrointestinal dysfunction [79,80,81], possibly resulting from reactivation-induced damage to ENS neurons or virus replication in the gut wall.

### 3.2. Entry of VZV into the Peripheral Nervous System

**Figure 2:**
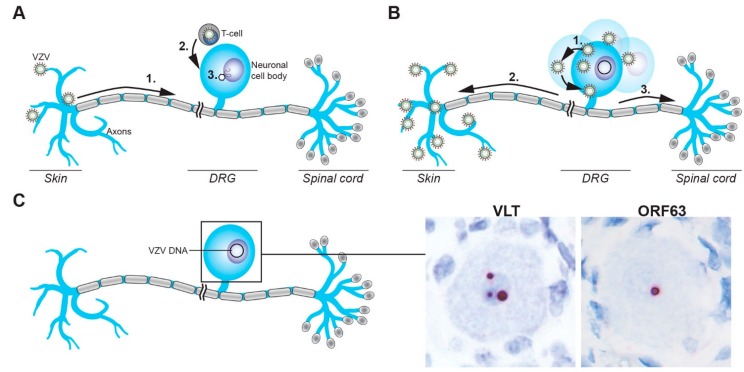
Schematic representation of the establishment of, and reactivation from, VZV latency in sensory neurons. (**A**) VZV gains access to sensory ganglion neurons via the infection of nerve endings in skin and retrograde axonal transport to the neuronal cell body (1) or direct infection of cell bodies via VZV-infected T-cells (2), followed by the release of the viral genome into the nucleus (3). (**B**) VZV reactivation results in virus replication and spread in the cell body (1), followed by transaxonal spread to the skin to cause HZ (2), possibly involving concordant virus spread to the spinal cord (3). (**C**) VZV latently infected sensory neurons contain viral episomal DNA in their nuclei and express *VLT* and/or *ORF63* RNA, as shown by RNA in situ hybridization on human TG (red signal). Magnification: 400× with 2.5× digital zoom.

Two non-mutually exclusive routes by which VZV may infect sensory ganglion neurons have been proposed (Figure 2A). First, VZV could enter nerve endings innervating the dermis at sites of cutaneous lesions and gain access to ganglia by retrograde axonal transport. This route is supported by the detection of viral antigens in Schwann cells and peripheral nerve axons in the dermis of varicella patients [82], and observations that HZ occurs at the site of varicella vaccine inoculation [83] or the sites most affected by varicella [4]. More recently, VZV infection of axons and retrograde axonal transport to neuronal cell bodies was formally demonstrated in cell culture [84,85], possibly involving fusion between VZV-infected non-neuronal cells and neuronal axons [86]. Notably, nerve endings are located in close proximity to the cutaneous vasculature at the dermal–epidermal junction and in hair follicles [87], supporting a model in which VZV may concurrently infect epidermal or hair follicle keratinocytes and neurons via local cell-to-cell spread. Second, VZV-infected lymphocytes, most likely T-cells, could disseminate the virus to ganglia during varicella-associated viremia. This is supported by detection of VZV DNA in ganglia obtained at autopsy from patients in the prodromal stage of varicella [7]. Moreover, localized injection of live-attenuated VZV vaccine virus results in the establishment of viral latency in bilateral DRGs and enteric ganglia [76]. VZV infects T-cells, prolongs their survival via STAT3 phosphorylation and induction of survivin [88], and modulates their phenotype to induce an activated skin-tropic memory T-cell [89,90,91]. While the prerequisites for T-cell entry into ganglia are unknown, intravenously injected VZV-infected tonsillar T-cells have been shown to transfer the virus to human foetal DRG xenografts implanted under the kidney capsule in severe combined immunodeficiency (SCID) mice [92]. Moreover, T-cells infected with the simian varicella virus (SVV), the closest relative to VZV and natural cause of varicella and HZ in non-human primates, has been detected in the ganglia of non-human primates during primary infection [93]. Thus, the definitive route(s) by which VZV infects ganglionic neurons, with or without cutaneous innervation, needs to be investigated in future studies.

## 4. Transcriptional Repression of Latent VZV Genomes

In the absence of robust animal models, VZV latency studies have been dominated by the use of naturally VZV-infected cadaveric human ganglia, either snap-frozen at autopsy [94] or explanted into short term cultures (reviewed in ref. [95]), although the latter is not reported to result in the production of infectious virions [96]. More recently, the use of in vitro hESC-derived neurons has provided a more accessible approach to latency studies [56,97], principally due to the ability to examine both the establishment of, and reactivation from, latency in sequential fashion over longer periods using mutant viruses and assays that are simply not compatible with wild type, VZV-infected, post-mortem ganglia.

### 4.1. VZV Transcription in Human Ganglia

The difficulty of obtaining and working with post-mortem human ganglia has resulted in discrepant numbers and identities of viral transcripts, and in some cases, the corresponding proteins have been detected [98,99,100,101,102]. The latter has been confounded by the use of ascites-derived murine and rabbit antibodies that also contain endogenous antibodies that react with human blood type A antigens expressed by sensory neurons [103,104]. Consequently, VZV protein expression in human ganglia appears to be absent or extremely rare and is most likely associated with de-repression of the latent viral genome or in the early stages of reactivation [98,100]. The role of cellular dysregulation following death is undoubtedly a major influence [105], reflected by the post-mortem interval (PMI) which is a major factor in determining the number and identity of viral transcripts present [106]. Most recently, unbiased virus nucleotide-enriched ultra-deep RNA-sequencing confirmed that viral transcription in short-PMI (<9 h post mortem) human ganglia is highly restricted and limited to *VLT*, frequently accompanied by co-expression of *ORF63* RNA, albeit at lower quantities than *VLT* [27,73,74,75] (Figure 2C). Interestingly, fewer TG neurons express *VLT* (on average, 0.49%) and *ORF63* (0.36%) by in situ hybridization than the reported 2–5% of neurons harboring latent VZV DNA [27], suggesting that not all VZV genomes present in TG neurons are transcriptionally active. Although both *VLT* and *ORF63* RNA retain the potential to be translated during latency, these viral proteins could not be detected in latently infected human ganglia by immunohistochemistry [27]. However, given the very low abundance of *VLT* and *ORF63* RNAs, alternative approaches with higher sensitivity, such as screening for the loading of *VLT* and *ORF63* RNA onto polysomes combined with targeted enrichment for viral RNAs could provide conclusive data. Thus, VZV latency in naturally infected human ganglia is characterized by the exclusive expression of *VLT* and often, *ORF63*.

### 4.2. Comparison of VZV Latency In Vivo and In Vitro

Although naturally VZV-infected, short-PMI, human cadaveric ganglia most closely resemble *in vivo* VZV latency, ganglia from these individuals are likely to have been latently infected for many decades. In contrast, when using in vitro hESC-derived neuronal models, latency is usually profiled within 14 days. Although transcriptome-wide profiling by RNA-sequencing of in vitro “latent” VZV infections has revealed a marked reduction in viral gene expression, all viral genes are expressed in the absence of detectable levels of viral proteins [56,97]. While limited enrichment for the classical VZV latency-associated *ORF63* RNA has been observed, it is not known whether these systems express detectable *VLT* and if so, whether the latent *VLT* isoform is transcribed [27,56,97]. This has led to the introduction of new terminology, such as non-productive or quiescent infections, and questions over the relative merits of in vitro models. As a counterpoint, these models have proven to be highly informative of early events during the VZV infection of neurons and the establishment of latency. Moreover, in vitro models remain the only system in which the establishment of latency and subsequent reactivation can be studied, with these studies, as a whole, benefitting increasingly from continual improvements in culture longevity and axonal infection protocols.

### 4.3. Epigenetic Silencing of the Latent VZV Genome

The molecular mechanisms by which VZV latency is established are largely unknown and much of what is assumed is actually informed by studies of the related herpes simplex virus type 1 (HSV-1). Here, the key concept is that during the traversal of neuronal axons, the genome-containing nucleocapsid is delivered to the nucleus in the absence of functional tegument proteins for IE gene transcription (i.e., VZV ORF10 protein—the homolog of HSV-1 virion protein 16 [VP16]), and this allows loading and maintenance of repressive chromatin upon the viral episome which inhibits viral transcription ([107] and reviewed in ref. [108]). Whether this is a rapid or slow process is not known, although data from VZV in vitro models may provide support for the latter, i.e., viral transcription in newly infected neurons is gradually suppressed over time, eventually leading to the transcriptional profile that we correlate with latency in human ganglia (i.e., the expression of *VLT* and frequently, *ORF63* RNA). Either way, the remarkable switch between lytic and latent transcriptional states is best explained by the assembly of repressive chromatins upon the viral episome. Indeed, studies using late PMI (>9 h post mortem) VZV-latently infected human ganglia have demonstrated euchromatic (H3K9ac) chromatin modifications on promoters of latency-associated *ORF63* and *ORF62*, the latter of which is frequently expressed in late PMI deregulated ganglia [109]. In contrast, promoters of viral genes, *ORF14* and *ORF36*, which are not expressed during latency, nor detected in late-PMI ganglia, have not been associated with H3K9ac [109]. The increase in viral transcription with increasing post-mortem intervals suggests that these studies need to be extended to shorter PMI and genome-wide analyses. Indeed, one might speculate that all but *VLT* and *ORF63* loci would be bound by repressive chromatin, and it will be important to determine whether the *VLT* locus remains active due to the presence of predicted sequence motifs for flanking chromatin insulators such as CCCTC-binding factor (CTCF), as has been observed for HSV-1 [110].

## 5. The Varicella Zoster Virus Latency-Associated Transcripts: *VLT* and *ORF63*

While all *alphaherpesviruses* express latency-associated transcripts (*LATs*), their size, coding potential, functions, and mechanisms of action may vary.

**Figure 3:**
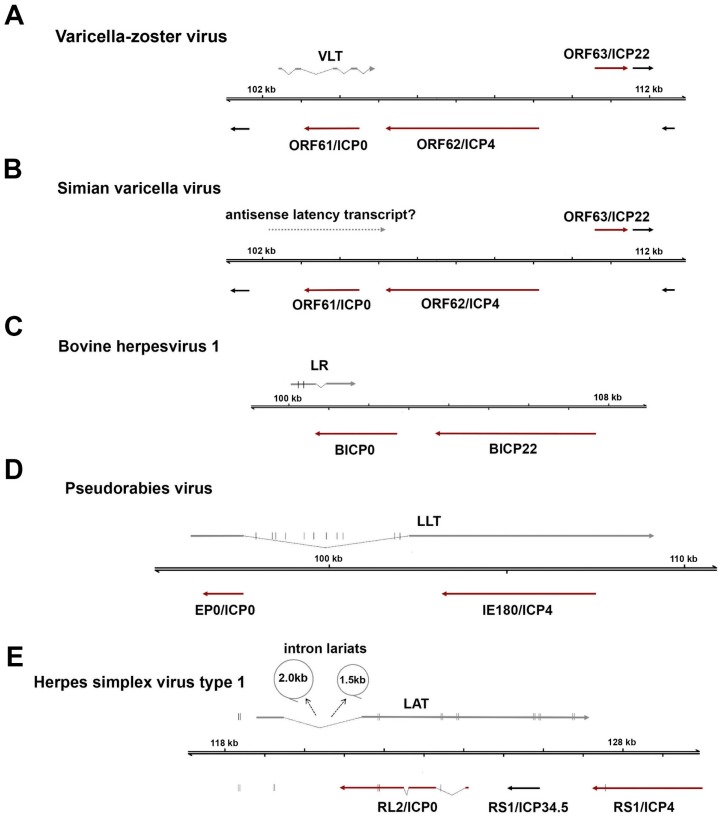
Comparison of latency-associated transcripts among *alphaherpesviruses*. All *alphaherpesvirus* latency-associated transcripts (*LATs*) are located antisense to the infected cell polypeptide 0 (ICP0) locus, encoding a conserved major immediate-early transactivator (ICP0 or homologs). (**A**) The VZV latency-associated transcript (*VLT*) is a 496-nucleotide, multi-exon mRNA that is partially antisense to the *ORF61* coding region via exons 3 and 4. (**B**) Transcripts mapping antisense to simian varicella virus *ORF61* are expressed during latency (dotted arrow), but their identity has not yet been defined. (**C**) Bovine herpesvirus 1 encodes a 2.2 kb latency-related (*LR*) RNA and encodes two miRNAs in exon 1 (**D**) The pseudorabies virus large latency transcript (*LLT*) is the largest characterized *alphaherpesvirus* latency transcript and encodes eleven distinct miRNAs within the spliced intron. (**E**) The 8.2 kb herpes simplex virus type 1 (HSV-1) *LAT* undergoes splicing which yields two highly stable intron lariats, approximately 1.5 and 2.0 kb in size (shown as circles). Note that latency transcripts are shown as grey arrows, immediate early viral transactivators as dark red arrows, and encoded miRNAs as short vertical lines. Scaling across all schematics is equal.

The identification of *VLT* represents a major advance in studies of VZV latency. *VLT* is encoded antisense to the *ORF61*, and it is remarkable to note that all known *alphaherpesvirus* latency-associated transcripts (*LATs*) originate from genomic regions encoding infected cell polypeptide 0 (ICP0) homologs (Figure 3). This is indicative of significant evolutionary conservation, although the encoding of *LATs* and ICP0 homologs antisense to each other makes it harder to assess their individual contributions toward the maintenance of this locus. Notably, VZV appears to be the only *alphaherpesvirus* that has been analysed in detail to date that consistently expresses an additional latency transcript, *ORF63*, in human TGs.

### 5.1. Comparison of VLT and LATs of Related Alphaherpesviruses

*VLT* is a polyadenylated RNA comprising at least five distinct exons and is encoded antisense to VZV *ORF61*, a homolog of the HSV *RL2* gene (encoding ICP0). During latency, a single *VLT* isoform is expressed in the neurons of virtually all analysed VZV-infected human TGs [27], although it is not known whether *VLT* is expressed in all VZV-infected neurons. *VLT* appears to be structurally complex compared to the *LATs* of HSV-1, pseudorabies virus (PRV) and bovine herpesvirus 1 (BHV-1), which are composed of only two exons and a single intron (Figure 3). The possible exception is the closest relative of VZV, the non-human primate SVV, which expresses a transcript mapping to the same region as *VLT* during latency [111,112], although more detailed studies are needed to determine the exact location and structure of the SVV *VLT* homolog. Unlike the stable *LAT* introns of HSV-1 that accumulate to high abundancies in latently infected human TG [27,113], *VLT* appears to be expressed at relatively few copies per neuron [27]. Furthermore, while the *LATs* of HSV-1, PRV, and BHV1 encode viral miRNAs [114,115,116], there is no evidence for the expression of VZV miRNAs encoded within *VLT*—or anywhere else in the VZV genomes—in latently-infected human ganglia [27].

Although *VLT* is the predominant transcript expressed during latency, its expression is not restricted to the latent phase. During VZV lytic infections of epithelial cell or melanoma cell cultures, multiple alternatively spliced *VLT* isoforms were identified [27]. Termed *VLT_ly_*, these lytic isoforms are transcriptionally more complex than the “core” latent *VLT* isoform detected during latency. Exon skipping, intron retention, and additional upstream exons are all features of *VLT_ly_*, with most variation apparently occurring between “core” exons 3 and 4. Notably, the core latent *VLT* isoform has never been observed in lytic infections which may suggest that the core isoform is driven by a cell type (i.e., neuron) specific promoter. During productive infections, *VLT_ly_* is transcribed with true-late kinetics, is defined as being absolutely dependent on viral replication, and is translated into proteins (pVLT) in cell cultures and zoster skin lesions [27]. Although the expression and function of *LATs* during lytic *alphaherpesvirus* infection is incompletely understood, the observed features of *VLT* are consistent with the general pattern of *LATs*. During lytic infection, *LATs* appear to be expressed with late kinetics [27,117]. Alternative *LAT* isoforms are produced using different transcription start sites or alternative splicing [27,118,119], and some *LATs* encode proteins [27,120]. Given that hESC neuronal models support both lytic and quiescent VZV infections [56,97], these could be particularly useful for investigating the regulation of lytic and latent isoforms of *VLT* in the same cell type.

### 5.2. Function(s) of VLT

The functional characterization of *VLT* during lytic and latent stages is now a priority. Early investigations using transfection assays showed that *VLT* selectively represses *ORF61* transcription when co-expressed with multiple VZV coding IE genes (*ORF61*, *ORF62* and *ORF63*) in ARPE-19 epithelial cell cultures, resulting in diminished expression of the ORF61 protein, IE61 [27]. This effect is not pVLT-specific as mutations of the initiating *ATG* start codon (to *ATA*) showed a similar effect—suggesting that it is mediated by the RNA itself. While the specific functions and requirements of *VLT* and *VLT_ly_* isoforms during lytic and latent infections remain unknown, these data allow us to speculate that *VLT* may function to maintain latency by repressing the transcription of *ORF61*, a promiscuous transcriptional regulator, during lytic infections. However, it will be pivotal to confirm these observations in other experimental systems, especially since not all functions attributed to *LATs* of related herpesviruses in vitro can subsequently be confirmed *in vivo* [121,122].

### 5.3. Function(s) of ORF63

**Figure 4:**
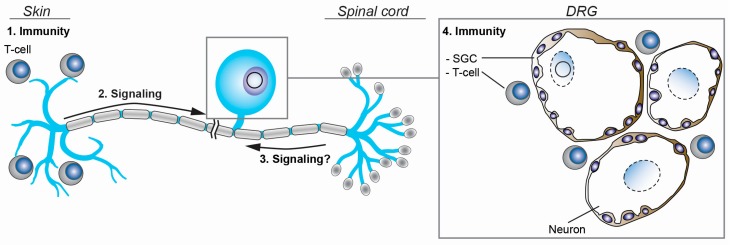
Schematic representation of the regulation of VZV latency and reactivation. The activation of neuronal signaling pathways in response to stimuli at the periphery (2) and possibly, within the spinal cord (3) may induce VZV reactivation. At the same time, adaptive T-cell-mediated immune responses in skin (1) and ganglia (4), and the local ganglionic innate immunity provided by neuron-interacting satellite glial cells (SGC) are believed to prevent symptomatic VZV reactivation.

VZV expresses two latency-associated transcripts, *VLT* and *ORF63* RNA, the expression of which correlates significantly in human TG, independent of the latent viral DNA load [27], suggesting that expression of both transcripts is linked. It is unclear whether *VLT* and *ORF63* RNA are produced by the same or by distinct populations of neurons, and how this may influence the ability of the virus to reactivate. *ORF63* is essential for VZV replication [123] and its encoded protein, IE63, functions not only as a transcriptional regulator activating E genes [124] but also modifies neuronal susceptibility to apoptosis and functions as a immunoevasin that blocks type I interferon (IFN) signaling [125,126,127]. As such, we speculate that *ORF63*, once translated (IE63), could be crucial for the initiation of reactivation, supported by the correlation between *ORF63* RNA abundance and PMI [106]. The observation that IE61 is required for nuclear entry of IE63 in guinea pig enteric neurons, yet to be determined in human neurons [128], suggests that *VLT*-mediated repression of *ORF61* transcription and translation may also function to retain IE63 in the cytoplasm and prevent the transactivation of lytic viral promoters (Figure 4A).

## 6. VZV Reactivation: Restarting Lytic Gene Expression

Symptomatic VZV reactivation, which leads to HZ and/or associated pathologies, typically occurs just once or twice in an infected individual’s lifetime and likely requires two events—stimulus of a latently-infected neuron and the overriding of existing immune protection mechanisms. In contrast, asymptomatic reactivation is thought to occur more frequently [2,129]. Possibly, the phase of intra-ganglionic virus replication that is presumed to occur upon VZV reactivation [130,131] (Figure 2B) before the virus descends down sensory axons to the skin provides the host immune system more time to control most reactivation events before becoming symptomatic. The environmental factors and molecular mechanisms that underpin VZV reactivation in humans remain poorly understood. Clinical observations of HZ after trauma [132] or neurosurgery [133,134] suggest that signalling events from the periphery may induce VZV reactivation in the soma of sensory neurons. Two main signalling pathways have been implicated in VZV reactivation: the phosphatidylinositol-3 kinase (PI3K)-Akt pathway and the mitogen-activated protein kinase (MAPK) pathway. The nerve growth factor (NGF) receptor, TrkA, is expressed by a subpopulation of sensory neurons and signals via the PI3K-Akt and MAPK pathways [135], and depletion of NGF from VZV-latently infected neurons can lead to the reactivation of VZV in some in vitro latency systems [56,97]. The role of NGF signalling in contributing to the maintenance of VZV latency has been confirmed in VZV-latently infected human TG removed within 24 h after death [136]. Intriguingly, the effect of chemical PI3K blockade on VZV reactivation depends on the experimental conditions, as PI3K inhibition did not reactivate VZV in ex vivo cultures of naturally infected human TG neurons [136] and variable effects have been obtained in hESC-derived neurons [56,97]. NGF depletion results in the phosphorylation and activation of the MAPK family member, c-Jun N-terminal kinase (JNK) and is critical for efficient VZV replication in hESC neurons. Selective JNK inhibition limits VZV reactivation in vitro, suggesting JNK signalling plays important role for VZV reactivation in neurons [137].

To reactivate from latency, the latent viral episome needs to be de-repressed, and viral gene expression has to occur as a downstream event of the PI3K-Akt and/or JNK pathways. The mechanisms underlying the reversal of repressive chromatin on the VZV genome and the viral gene expression machinery required for VZV reactivation are poorly understood. Unlike HSV-1, the inhibition of histone deacetylases resulted in failed production of the infectious progeny virus [56], but led to viral DNA replication and expression of late genes in vitro [97]. It may be that additional chromatin modifications, like the methylation of histone H3 lysine 4 (H3K4) or demethylation of H3K9, need to take place before VZV reactivation can occur [138]. Alternatively, JNK signalling may induce a methyl/phospho switch on promoters of VZV lytic genes that facilitates their expression and virus reactivation as observed during HSV-1 latency and reactivation [139]. Clearly, additional environmental factors and neuronal signalling pathways are involved, and the overall outcome of VZV infection will be determined by the combined integration of all these pathways.

## 7. Intrinsic, Innate, and Adaptive Immunity to VZV Infection in Ganglia

The intrinsic properties of neurons and the innate and adaptive immune responses mounted by ganglion-resident or infiltrating cells that contribute to the control of VZV replication in ganglia are unknown. VZV infection of human foetal DRG xenografts in SCID mice produces transient virus replication and spread, followed by persistently lower levels of viral DNA and limited viral transcription after 4–8 weeks [92], suggesting that adaptive immune responses are not essential for the control of VZV replication in this model system. Intrinsic differences in neuronal subpopulations affect the outcome of VZV infection, as virus replication is blocked in neurons expressing the mechanoreceptive marker RT97, but not in neurons expressing the nociceptive marker peripherin [140]. However, it is not clear which neuron subtype(s) harbor latent VZV in naturally infected humans and whether all neurons are equally capable of supporting VZV reactivation.

### 7.1. Satellite Glial Cells and Innate Immunity

Although sensory neurons produce little type I IFN α/β, neurons are sensitive to IFN-α/β signalling, as the exposure to IFN-α/β at nerve endings prevents retrograde axonal transport of *alphaherpesvirus* virions [141]. Notably, various cytokines/chemokines are produced in VZV-infected foetal DRG, including IFN-α, interleukin 1 alpha (IL-1α), IL-6, CXCL10, and transforming growth factor beta [140]. While the cellular source of these cytokines is unknown, satellite glial cells (SGC) are prime candidates. SGC are a specialized ganglionic cell type that shares phenotypic and functional features with professional antigen presenting cells like macrophages and dendritic cells [142,143]. SGC completely enwrap the neuronal cell body and provide physical and nutritional support to the neuron, but also function as tissue-resident innate immune cells that express pattern recognition receptors (e.g., Toll-like receptors), phagocytose, produce inflammatory mediators and potentially regulate local T-cell responses [142,143,144]. Moreover, SGC surrounding HSV-1 infected neurons produce IL-6 and tumour necrosis factor α [145], and we have shown that SGC are activated in response to primary SVV infection in monkeys [146]. However, more detailed studies on the role of SGC in response to ganglionic VZV infected are warranted.

### 7.2. T-Cell Immunity

VZV reactivation, clinically presenting as HZ, is associated with the waning of systemic VZV-specific T-cell immunity, but not humoral immunity, suggesting a critical role of VZV-specific T-cell memory in preventing virus reactivation [147,148]. However, a large fraction of pathogen-specific memory T-cells are retained in organs, referred to as tissue-resident memory T-cells (T_RM_), and provide swift protective immunity upon pathogen re-exposure [149,150]. In analogy to HSV-1 mouse models [151,152,153,154] and human TG [155], VZV-specific T_RM_ are expected to home to sites of latency and reactivation in ganglia and skin. Indeed, recent studies showed that VZV-specific T_RM_ cells persist in the healthy skin of latently VZV-infected adults decades after primary infection [156]. Profound T-cell responses detected in ganglia of HZ patients hint at the presence of VZV-specific T_RM_ in ganglia [157,158]. While VZV-specific T-cells in latently infected human TG have not previously been detected, this study analysed only a restricted panel of VZV proteins (IE4, ORF29 protein, IE62, and IE63) [159]. More detailed studies are needed to determine the presence, specificity, and function of VZV-specific T_RM_ in latently VZV-infected human ganglia in regard to their contribution to VZV latency and reactivation control. This will provide more insight into the inverse relationship between VZV-specific T-cell immunity and the risk of HZ development.

## 8. Future Perspectives

VZV has evolved with the ability to establish lifelong latent infection in neurons, enabling the virus to reactivate later in life and cause recrudescent disease accompanied by VZV spread to naïve individuals. Throughout the decades of latency, the virus must ensure survival of the infected neuron, avoid irreversible silencing of the viral episome, and prevent elimination by host immune responses. Although the mechanisms that coordinate the interplay between the virus and the host remain largely unknown, the many recent exciting advances in the field provide us with the questions to be addressed and tools (ex vivo and in vitro latency models) that can be used to answer them. Thirty-five years on from the discovery of latent VZV residing in sensory ganglia [5], the identification of *VLT* and the latent VZV transcriptome in naturally-infected, short-PMI TG has provided us with an new perspective on VZV latency [27]. With this advancement, we also propose to enhance the definition of VZV latency as requiring (i) the presence of the viral genome as an episome in the nucleus of host cells without production of infectious progeny, (ii) the capacity of latent VZV to reactivate and produce infectious virus, and (iii) a distinct and restricted pattern of viral gene transcription, i.e., exclusive expression of *VLT* and/or *ORF63* RNA.

It is now critical to determine the roles of (and potential interplay between) *VLT* and *ORF63* RNA in latently infected short-PMI human TG [106] and to analyse their functions in the establishment of quiescence in human pluripotent stem cell (hPSC; hESC/hiPSC)-derived neuronal models [56,97,160,161] or in the reactivation from latency using these same models and/or ex vivo cultures of dissociated naturally VZV-infected human ganglia [136]. Human TG are composed of diverse subtypes of neurons [162], and not all neuronal subpopulations may be equally susceptible to VZV infection [140], as has been shown for HSV-1 [163,164]. Therefore, in situ analyses are required to determine whether *VLT* and *ORF63* RNA are co-expressed by the same or distinct neurons and to define the subtypes of neurons harbouring the latent VZV genome. Recent developments in the generation of recombinant VZV (reviewed in ref. [53]) will facilitate the functional analysis of *VLT*/pVLT and *ORF63* RNA/IE63 using hPSC-derived neuronal systems. Current evidence suggests that both pVLT and IE63 are not expressed, or are expressed below detectable levels, in latently infected ganglia [27,103,104]. While we have identified one possible function of the *VLT* RNA [27], no functions are currently attributed to *ORF63* RNA or pVLT.

Transcriptional regulation of VZV gene expression during lytic and latent infection has been poorly defined. In analogy to HSV-1 [165], the reactivation of latent VZV is presumed to follow a regulated cascade of gene expression, and more detailed analyses of lytic VZV transcriptional regulation are warranted to provide insight into the requirements of VZV reactivation. Recent developments in next-generation sequencing have facilitated the detection of low-abundant VZV genomes in latently infected human TG [58,59] and will open up new avenues to unravel epigenetic modifications of latent VZV genomes using chromatin immunoprecipitation sequencing (ChIP-seq) for histone modifications or CTCF binding. Furthermore, in vitro quiescence models could be used to prospectively map epigenetic chromatin modifications on the VZV genome during quiescent and reactivated stages of infection. Of particular interest is also be the relative expression levels of *VLT* and *ORF63* RNA, and IE63 and IE61 during the initiation of virus reactivation.

The factors triggering and barriers restricting VZV reactivation are poorly understood and are of great interest for future research. The infrequent clinical reactivation of latent VZV suggests that either virus reactivation is rare or that most reactivation events are cleared before HZ can develop. Local immune responses mediated by VZV-specific T_RM_ in skin [156,166] or ganglion, or neuron-interacting SGC [143,144] are likely to be pivotal factors controlling VZV reactivation, e.g., by secreting IFNs to block early reactivation attempts [167]. The recently developed in vitro and ex vivo models to study VZV reactivation [56,97,136,160,161] facilitate systematic analysis of the signalling pathways modulating viral latency, and could be used to investigate the roles of non-neuronal cells and their secreted antiviral factors in this process.

Post-mortem acquired human samples are extremely informative if handled carefully, especially for investigating human-restricted pathogens like VZV, but they are also restrictive in that the resulting datasets only represent a “snapshot” of latency. To overcome this, we hope that technological advances in specific subtypic differentiation methodology for human sensory/sympathetic neurons or organoid culture system from hPSC combined with single cell sequencing methodologies targeting transcriptomes and epigenomes will provide novel ways to examine the dynamics of VZV latency and provide new insight into the molecular mechanisms that control this.

Finally, the discovery of *VLT* and its potential role in governing VZV latency and/or reactivation also opens up the tantalising possibility of re-engineering current VZV vaccines not to establish latency or reactivate. While a significant amount of work is required to get from here to there, not least determining whether *VLT* is truly required for latency and/or reactivation, pursuing this long term goal gives new impetus to the VZV research field.
